# Case Report: Generalized anxiety disorder and hypertension: a bidirectional loop unraveled by integrated management

**DOI:** 10.3389/fpsyt.2025.1600910

**Published:** 2025-10-02

**Authors:** Junming Chen, Yuxing Yang, Fugui Jiang

**Affiliations:** Sichuan Provincial Center for Mental Health, Sichuan Provincial People’s Hospital, School of Medicine, University of Electronic Science and Technology, Chengdu, China

**Keywords:** generalized anxiety disorder, hypertension, bidirectional relationship, multimodal therapy approach, neurobiological pathway

## Abstract

**Background:**

Generalized anxiety disorder (GAD) and hypertension (HTN) exhibit a clinically significant bidirectional relationship characterized by neuroendocrine dysregulation and autonomic dysfunction. Their comorbidity presents diagnostic and therapeutic challenges due to overlapping symptoms and fragmented care pathways.

**Case presentation:**

We report a 61-year-old male with 26-year refractory HTN and new-onset GAD triggered post-thyroidectomy. Despite triple antihypertensive therapy (nifedipine, arotinolol, sacubitril/valsartan), blood pressure (BP) remained uncontrolled (176/105 mmHg) with severe anxiety (HAMA = 36). Secondary HTN investigations were negative. Multimodal management combining pharmacotherapy (escitalopram, tandospirone), transcranial magnetic stimulation (9 sessions), biofeedback (14 sessions), psychotherapy, and lifestyle interventions achieve: mean BP decreased significantly from 176/105 mmHg to 125/72 mmHg during hospitalization; significant anxiety reduction (HAMA = 3), mean BP stabilized at 131/77 mmHg with 50% reduction in antihypertensive dosages, normalization of elevated ACTH (99.9→normal pg/mL) and cortisol (18.7→normal μg/dL) and sustained improvement at 6-month follow-up.

**Conclusion:**

This case demonstrates thyroidectomy-induced endocrine disruption as a novel trigger in the GAD-HT bidirectional loop. Multimodal therapy targeting shared neurobiological pathways (HPA axis, autonomic regulation, serotonin signaling) effectively breaks this cycle, underscoring the imperative for integrated mental-cardiovascular care in treatment-resistant cases.

## Introduction

Hypertension (HTN) and generalized anxiety disorder (GAD) represent a significant bidirectional comorbidity with profound global health implications. HTN affects approximately 32% of adults worldwide, driven by genetic predisposition, lifestyle factors (e.g., high sodium intake, physical inactivity), and chronic stress exposure (1). Concurrently, GAD—characterized by persistent, excessive worry—has a global prevalence of 7.3% and demonstrates frequent comorbidity with cardiovascular conditions (2). Critically, these disorders engage in a self-perpetuating cycle: chronic anxiety exacerbates HTN through sympathetic nervous system hyperactivity and hypothalamic-pituitary-adrenal (HPA) axis dysregulation, while uncontrolled HTN amplifies psychological anxiety through physiological burden and illness-related worry (3). Notably, research has established a significant causal effect of anxiety on hypertension risk (4).

This pathophysiological interplay involves interconnected mechanisms. Neuroendocrine dysregulation—particularly involving the hypothalamic-pituitary-adrenal (HPA) axis—increases catecholamine release (5) and elevates cortisol levels thereby promoting vasoconstriction and endothelial dysfunction (6). Concurrent autonomic imbalance directly disrupts blood pressure (BP) regulation while exacerbating anxiety symptoms (7). Critically, autonomic dysfunction reduces heart rate variability (HRV) (8), which mediates 33–80% of anxiety’s effect on hypertension through altered baroreflex sensitivity (9). Furthermore, anxiety impairs self-care behaviors (e.g., medication adherence, dietary control) via maladaptive coping strategies (3, 10). Additionally, endocrine dysregulators such as thyroid hormone (TH) abnormalities can concurrently modulate vascular tone and anxiety pathways through adrenergic receptor upregulation and neurotransmitter alterations (11, 12). Collectively, these mechanisms drive persistent hypertension.

Clinically, this comorbidity presents substantial diagnostic and therapeutic challenges. Symptom overlap (e.g., palpitations, fatigue) obscures etiology, while pharmacotherapeutic interactions complicate management. Selective serotonin reuptake inhibitors (SSRIs), first-line GAD treatments, exert complex vascular effects through serotonin (5-HT) receptor modulation—where abrupt discontinuation may precipitate BP instability (13). Moreover, physiological stressors like thyroid dysfunction can trigger both conditions (14, 15). Current guidelines lack consensus on integrated approaches, often resulting in fragmented care between cardiovascular and mental health providers (16, 17).

This case report addresses this gap by examining a paradigmatic presentation of GAD and HTN exacerbated post-thyroidectomy. We elucidate three novel aspects:

1. The role of thyroidectomy-induced endocrine disruption as a bidirectional trigger;
2. Serotonin-mediated vascular modulation in SSRIs discontinuation phenomena;
3. Transcranial magnetic stimulation and biofeedback as a mechanism-specific intervention.

Through this lens, we demonstrate how multimodal management targeting shared neurobiological pathways can disrupt the GAD-HTN cycle, providing a framework for personalized comorbidity management.

## Case presentation

### History of illness

A 61-year-old male was diagnosed with recurrent HTN 26 years ago. He was prescribed nifedipine and metoprolol, which stabilized his BP at around 130/80mmHg. Despite taking his antihypertensive medication regularly, his BP would spike to over 200/100mmHg when he felt nervousness, leading to symptoms of dizziness and fatigue. Upon the advice of a psychiatrist, he started taking fluoxetine 20mg daily, which gradually helped lower his BP in conjunction with his antihypertensive drugs. However, he abruptly stopped taking fluoxetine a year later without any apparent reason. This led to increased nervousness and ineffective control of his BP with his antihypertensive medication.

The patient had renal sympathetic nerve ablation 7 years ago and is currently taking nifedipine 30 mg twice daily, arotinolol 10 mg twice daily, and sacubitril/valsartan 100 mg twice daily. He reported that his BP ranged from 140-170/80–100 mmHg when he was emotionally unstable and nervousness, and 140-150/80–90 mmHg when he felt relaxed during treatment with these antihypertensive medications.

**Figure 1:**
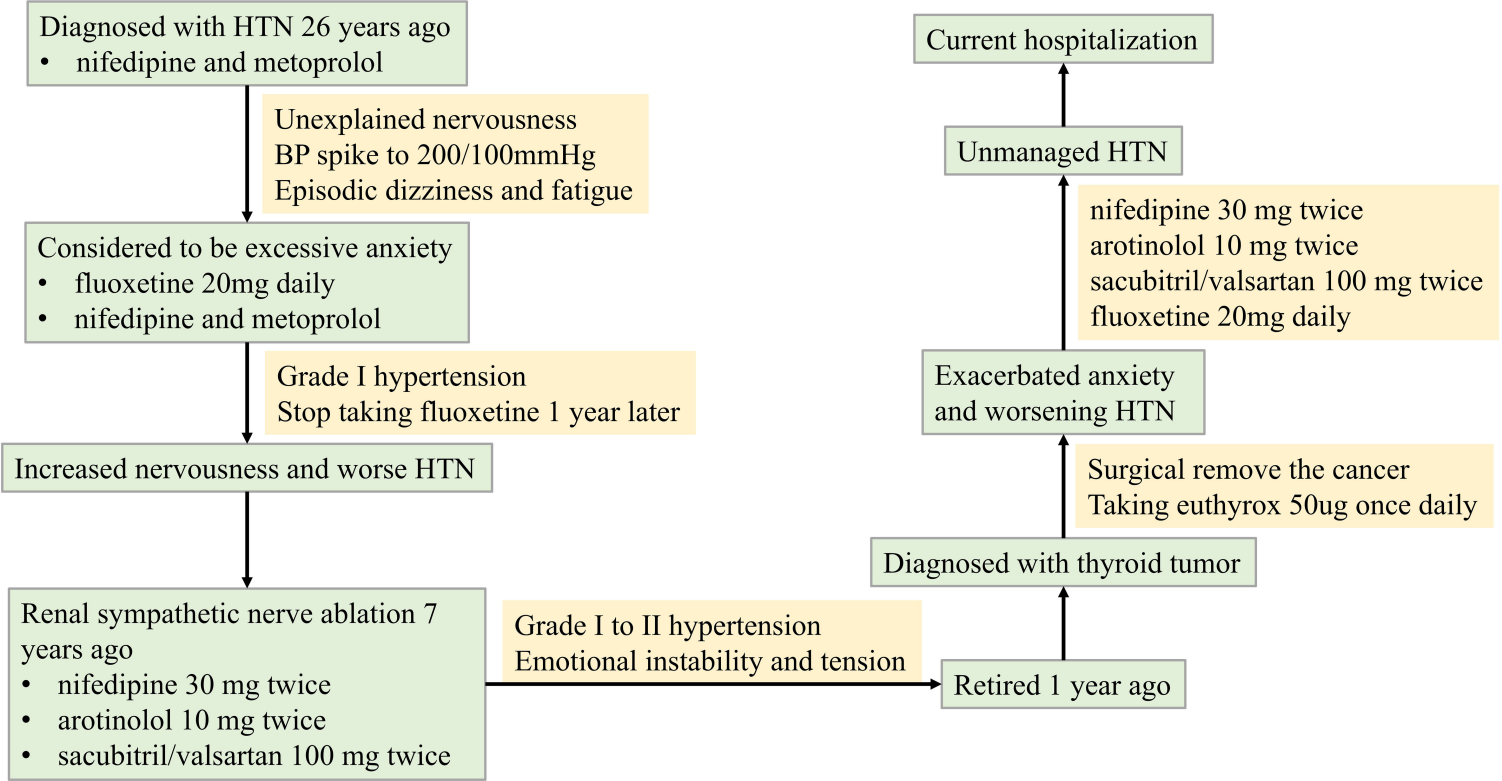
Timeline of history of illness.

This patient, who had retired one year prior, was diagnosed with a thyroid tumor and subsequently underwent thyroidectomy. Postoperatively, levothyroxine replacement therapy (50 μg daily) was initiated. He subsequently developed significant health-related anxiety, manifesting as severe nervousness, dizziness, insomnia, fatigue, and appetite loss. Consequently, fluoxetine (20 mg daily) was prescribed. Despite this intervention, his HTN became increasingly refractory to control. After one month of fluoxetine treatment without adequate HTN management, he required hospitalization. A timeline of the illness course is provided in **Figure 1**.

### Medical history

He has type 2 diabetes. Both parents and younger brother had HTN, and his father died of a stroke while his mother died of cerebral hemorrhage.

### Hospitalization treatment and follow-up

Despite undergoing comprehensive evaluation for secondary HTN during hospitalization, including renal artery ultrasound, aldosterone/renin and sleep apnea screening, all test results were negative. Moreover, the patient’s HTN remained refractory to antihypertensive therapy with a triple oral regimen consisting of nifedipine, arotinolol, and sacubitril-valsartan. Episodes of severe hypertension necessitated intravenous nitroglycerin to lower blood pressure, which further heightened the patient’s nervousness and anxiety. Subsequent psychiatric evaluation diagnosed GAD.

Initial psychiatric assessment indicated severe anxiety and moderate-to-severe depression. The patient scored 36 on the Hamilton Anxiety Rating Scale (HAMA), 32 on the Hamilton Depression Rating Scale (HAMD), 74 on the Self-Rating Anxiety Scale (SAS), and 63 on the Self-Rating Depression Scale (SDS) (**Table 1**). Endocrine laboratory findings included: Adrenocorticotropic hormone (ACTH) level of 99.9 pg/mL (normal range: 7.2–63.3 pg/mL), cortisol level of 18.7 µg/dL (normal range: 6.02–18.4 µg/dL), and glycated hemoglobin (HbA1c) of 6.36% (normal range: 4–6%). Thyroid function tests revealed an elevated free thyroxine (FT4) level of 26.2 pmol/L (normal range: 12.0–22.0 pmol/L) and a suppressed thyroid-stimulating hormone (TSH) level of 0.07 mIU/L (normal range: 0.27–4.20 mIU/L). HRV analysis demonstrated reduced overall autonomic nervous system activity, relative parasympathetic predominance, and impaired sympathovagal balance (LF/HF=0.458).

**Table 1:** Psychiatric evaluation during hospitalization and follow-up.

Time	HAMA	HAMD	SAS	SDS	SCL-90
1^st^ day of hospitalization	36	32	74	63	254
2-week of treatment	7	7	58	50	230
1-month follow-up	6	4	45	47	214
3-month follow-up	4	4	37	35	135
6-month follow-up	3	2	36	32	127

HAMA score: <7: no significant anxiety; 8-14: mild anxiety; 15-23: moderate anxiety; 24-56: severe anxiety.

HAMD score: <8: no depression; 8-16: mild depression; 17-23: moderate depression; ≥24: severe depression.

SAS score: <50: normal range; 50-59: mild anxiety; 60-69: moderate anxiety; ≥70: severe anxiety.

SDS score: <53: normal range; 53-62: mild depression; 63-72: moderate depression; ≥73: severe depression.

SCL-90 score: <160: within normal limits; 160-250: mild psychological distress; 251-350: moderate psychological distress; >350: severe psychological distress.

**Figure 2:**
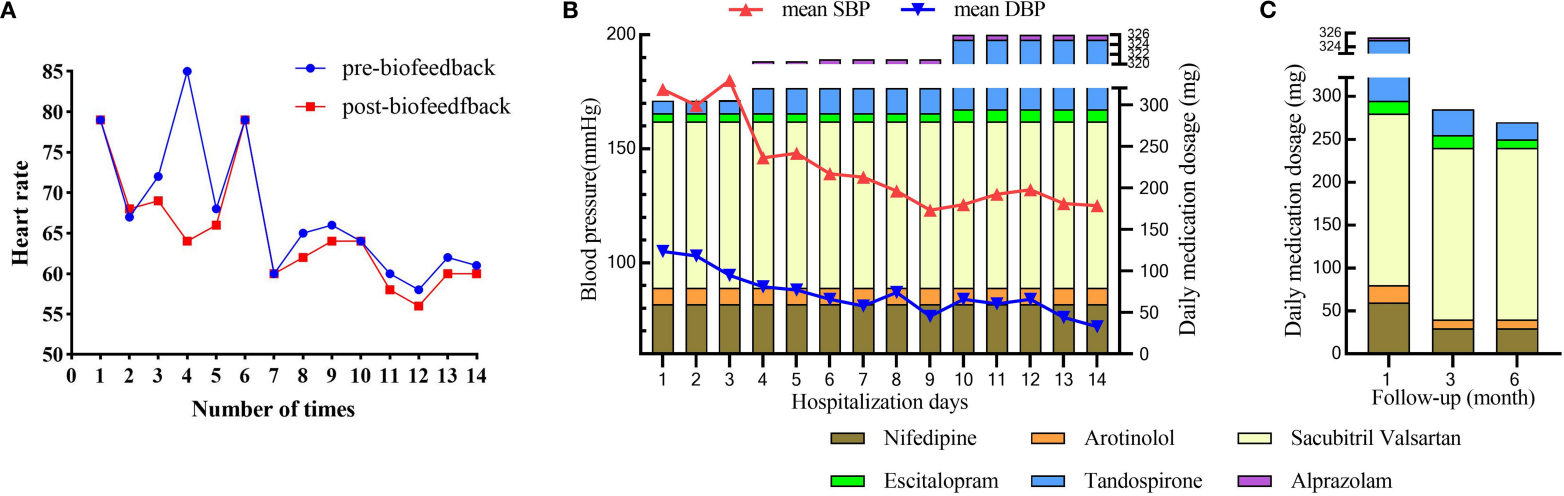
The outcome after multimodal therapy during hospitalization and follow-up. **(A)** Changes in heart rate during the period of biofeedback therapy; **(B)** Changes in mean systolic blood pressure (SBP) and diastolic blood pressure (DBP) alongside daily medication dosages over 14 days of hospitalization; **(C)** Changes in daily medication dosage over a 6-month follow-up period.

During the two-week hospitalization, the patient achieved significant improvement in anxiety and depressive symptoms through combined pharmacotherapy (escitalopram, tandospirone, alprazolam) and multimodal neuromodulation which comprised transcranial magnetic stimulation (TMS), biofeedback, group psychotherapy, individual psychotherapy, and disease-specific health education (**Table 2**). The dosages of oral medication and ambulatory mean BP during hospitalization was in **Supplementary Table S1**. Physiological parameters monitored during biofeedback sessions including *f*rontal surface electromyogram (sEMG), heart rate (HR) and skin temperature progressively normalized (**Table 3**). Notably, HR stabilized after seven biofeedback session (**Figure 2A**). Post-treatment psychometric scores decreased to HAMA = 7, HAMD = 7, SAS = 58 and SDS = 50 (**Table 1**). Concurrently, hypertension control substantially improved despite maintain the same oral antihypertension regimen. Mean BP decreased significantly from 176/105 mmHg to 125/72 mmHg (**Figure 2B**). discharge psychiatric evaluation indicated minimal residual anxiety and resolution of depressive symptoms to a mild level (**Table 1**). By discharge, ACTH and cortisol levels had normalized.

**Table 2:** The neuromodulation approaches for this patient.

Neuromodulation approach	Protocol		Number of times
TMS	Target	Right DLPFC (MIN: x=38, y=44, z=32)	9
	Pulse	Figure-8 Coil Magnetic Stimulator, Model YRD CCY-II (YIRUIDE Co., Ltd., Wuhan, China). Base frequency: 1Hz Sequence: 10 pulse/train, inter-train interval: 2000ms Total pulse/session: 1400	
	Stimulation intensity	80-120% MT. MT determination: contralateral APB muscle MEP≥50μV (5/10 trials). Actual mean: 108.3% ± 5.7% MT	
	Treatment course	Single session duration: 28.0±0.5 minutes (including calibration) Treatment frequency: 4-5 sessions/week Total number of sessions: 9 sessions (over 2-3 weeks)	
Biofeedback therapy	Equipment	Spirit-8 multiparameter biofeedback device (BioCom Technologies, USA). Synchronous acquisition of: EEG (Cz/Fz leads, International 10-20 System), fingertip skin temperature, ECG (Heart Rate Variability), and surface EMG (Electromyogram). Signals undergo real-time feedback after 256-bit A/D conversion.	14
	Training protocol	5-minute baseline assessment. 20-minute threshold training (Objective: frontal sEMG↓, HR↓, skin temperature↑). 5-minute recovery.	
	Implementation	Sessions conducted once daily for 7 consecutive days (total of 14 sessions). Strict maintenance of temperature at 22°C (± tolerance if applicable) and humidity of 50% RH. Electrode impedance: <5 kΩ.	
Group Psychotherapy	Mindfulness-based stress		3
	Reduction breathing relaxation training		3
	Cognitive-behavioral therapy		3
Individual psychotherapy			1
Disease-health education			1

TMS, Transcranial Magnetic Stimulation; DLPFC, Dorsolateral Prefrontal Cortex; MNI, Montreal Neurological Institute; MT, Motor Threshold; APB, Abductor Pollicis Brevis; MEP, Motor Evoked Potential; EEG, Electroencephalogram; ECG, Electrocardiogram; EMG, Electromyogram; A/D, Analog-to-Digital; sEMG, surface Electromyogram; HR, Heart Rate; RH, Relative Humidity.

**Table 3:** The outcome of biofeedback therapy.

Parameters	Pre-treatment	Post-treatment	P value
Frontal sEMG	403.7±31.5	397±33.2	<0.001
HR	67.4±7.9	64.8±6.7	0.001
ST	31.0±1.7	32.1±2.7	<0.001

sEMG, surface Electromyogram; HR, Herat Rate; ST, Skin Temperature.

Over the six-month follow-up period, the patient consistently adhered to a heart-healthy lifestyle, including a low-sodium, low-fat diet and regular aerobic exercise (cycling or swimming). His anti-anxiety medications were tapered, with only escitalopram and tandospirone continued (**Supplementary Table S2**). Thyroid function remained stable under levothyroxine supplementation (50 μg daily), FT4 levels between 16 and 19 pmol/L and TSH levels between 0.28 and 2.6 mIU/L. Psychiatric assessments indicated progressive improvement in anxiety and depression, with corresponding scores HAMA = 3, HAMD = 2, SAS = 36, and SDS = 32 (**Table 1**). Concurrently, hypertension control substantially improved; mean blood pressure (BP) stabilized at 131/77 mmHg, enabling a 50% dosage reduction in both nifedipine and arotinolol (**Figure 2C**).

## Discussion

This paradigmatic case elucidates the complex bidirectional pathophysiology linking GAD and HTN, demonstrating the critical importance of integrated management. Our patient’s 26-year history of recurrent HTN, exacerbated acutely post-thyroidectomy and accompanied by newly diagnosed severe GAD, presented a therapeutic challenge that was only resolved through combined psychiatric and cardiovascular intervention. The significant improvement in both anxiety symptoms (HAMA reduced from 36 to 7 during hospitalization; stabilized at 3 at 6 months) and HTN control (mean BP decreased from 176/105 mmHg to 125/72 mmHg during hospitalization; stabilized at 131/77 mmHg on reduced antihypertensive doses long-term) following targeted neuromodulation and pharmacotherapy underscores the interplay between these conditions.

The abrupt discontinuation of fluoxetine (20mg daily) one year prior highlights a critical clinical consideration. SSRIs exert complex vascular effects through serotonin (5-HT) receptor modulation, particularly involving vasoconstrictive 5-HT2A receptors. Abrupt cessation likely contributed to anxiety relapse and BP instability via dual mechanisms: peripheral withdrawal directly altering vascular reactivity and central serotonin depletion exacerbating anxiety, thereby increasing sympathetic tone (18, 19). This observation aligns with evidence linking SSRIs discontinuation syndrome to both psychological symptom rebound and cardiovascular dysregulation (20).

Post-thyroidectomy endocrine disruption emerged as a significant bidirectional trigger. The patient developed profound health-related anxiety alongside recurrent HTN following surgery. Laboratory findings revealed transient iatrogenic hyperthyroidism (TSH 0.07 mIU/L, FT4 26.2 pmol/L at admission), subsequently stabilized with levothyroxine (TSH 0.28-2.6 mIU/L, FT4 16–19 pmol/L at follow-up). Thyroid hormone (TH) dysregulation likely exacerbated both conditions: TH excess can upregulate β-adrenergic receptors, increasing sympathetic tone and vascular resistance (21), while simultaneously disrupting neurotransmitter systems (e.g., serotonin, dopamine) critical for mood regulation (22, 23). This TH instability, interacting with the underlying GAD-HTN pathophysiology, created a self-perpetuating cycle of physiological stress and psychological distress.

Concurrent HPA axis hyperactivity, evidenced by markedly elevated admission ACTH (99.9 pg/mL) and cortisol (18.7 μg/dL) levels (normalizing after integrated treatment), represents a core neuroendocrine mechanism in GAD-HTN comorbidity (24). The HRV profile observed in this patient—characterized by globally reduced variability, paradoxical relative parasympathetic predominance, and a markedly low LF/HF ratio (0.458)—presents a critical neurobiological signature of the GAD-HTN bidirectional loop (9). While autonomic dysfunction typically manifests as sympathetic hyperactivity in HTN or GAD individually (25, 26), this specific HRV pattern suggests a maladaptive compensatory response: chronic sympathetic overdrive may have exhausted autonomic reserves, leading to blunted overall HRV and ineffective parasympathetic counter-regulation. To simultaneously address neuroendocrine dysregulation and autonomic nervous system (ANS) imbalance, we developed a multimodal therapeutic protocol for this patient.

First, the biofeedback can directly address the identified ANS imbalance (27). Using synchronous multi-parameter feedback (sEMG, HR, skin temperature), we trained the patient to consciously modulate autonomic output, increasing skin temperature while reducing frontal muscle tension and HR. This precision targeting of the specific ANS dysfunction revealed by admission HRV—conducted over 14 intensive sessions—likely contributed significantly to the rapid stabilization of both BP and anxiety symptoms during hospitalization and the sustained improvement at follow-up.

Second, our neuropharmacological strategy demonstrates rational synergy with neuromodulation to restore autonomic homeostasis.​​ The medication regimen—escitalopram (a SSRIs) and tandospirone (a 5-HT1A receptor partial agonist)—were carefully selected not only for anxiolytic efficacy but also for their favorable autonomic profiles. Escitalopram minimally impacts cardiac conduction and may improve HRV over time through reduced central anxiety drive (28, 29). Tandospiron’s action on presynaptic 5-HT1A autoreceptors in the dorsal raphe nucleus modulates downstream projections to brainstem autonomic centers, potentially dampening excessive sympathetic outflow (30). Crucially, this pharmacotherapy complemented rather than replaced neuromodulation. The TMS protocol targeted the right dorsolateral prefrontal cortex (DLPFC), a key node in the cortical inhibition pathway over limbic and autonomic hyperactivity (31). Low-frequency (1Hz) stimulation to the right DLPFC likely enhanced inhibitory control, thereby reducing amygdala-driven sympathetic activation and facilitating the ANS retraining achieved through biofeedback.

This integrated “top-down” (TMS modulating cortical control) and “bottom-up” (biofeedback training peripheral ANS responses) approach, pharmacologically supported, represents a significant advance over isolated pharmacological or device-based interventions commonly reported. The resolution of HPA axis markers alongside clinical improvement supports their role in sustaining the comorbid cycle.

Finally, we believe that this multimodal strategy—TMS modulates cortical excitability in anxiety-relevant prefrontal circuits (32), sacubitril-valsartan concurrently enhances natriuretic peptides and inhibits the renin-angiotensin-aldosterone system (RAAS) (33), complemented by pharmacotherapy (escitalopram, tandospirone), biofeedback, psychotherapy, lifestyle modification, and endocrine management—successfully disrupted the GAD-HTN feedback loop. The progressive improvement across all metrics (anxiety scales, BP, endocrine markers, HRV) during hospitalization and sustained at 6-month follow-up validates this integrated approach.

## Conclusion

This case demonstrates a successful multimodal, mechanism-targeted approach for managing treatment-resistant GAD-HTN comorbidity. Key elements include vigilance regarding SSRI discontinuation effects, recognition of endocrine triggers (e.g., post-surgical thyroid dysfunction), biomarker-guided therapy (HPA axis hormones, HRV, thyroid function), and synergistic neuromodulation-biofeedback interventions.

Current literature predominantly documents ANS improvements with single-modality interventions (e.g., SSRIs or cognitive-behavioral therapy or exercise (17, 34, 35), while internationally endorsed guidelines for treatment-resistant anxiety disorders remain lacking (16). ​Critically, this case establishes the therapeutic efficacy of multimodal intervention—combining pharmacotherapy, targeted TMS, biofeedback, psychotherapy, and lifestyle modification—for severe autonomic dysfunction in treatment-resistant GAD-HT comorbidity. This integrated multimodal approach—simultaneously addresses all tiers of the “neuro-cardio-endocrine axis” —disrupts the pathological cycle typically resistant to conventional fragmented treatment modalities.

Future research should investigate the biomarkers like HRV parameters and HPA axis markers (ACTH/cortisol) for predicting treatment response in this comorbid population. Prospective randomized controlled trials should also be implemented to definitively compare the efficacy of our specific regimen (SSRIs + targeted TMS + biofeedback) against standard care or less intensive interventions. Addressing these key areas is essential to refine targeted interventions and improve outcomes for this challenging patient population.

## Data Availability

The original contributions presented in the study are included in the article/**Supplementary Material**. Further inquiries can be directed to the corresponding author.
