# Supplementary material for: Case Report: Generalized anxiety disorder and hypertension: a bidirectional loop unraveled by integrated management
Source: Front Psychiatry. 2025 Oct 2;16:1600910. doi: 10.3389/fpsyt.2025.1600910 (PMC12528110; doi:10.3389/fpsyt.2025.1600910)
Supplement: Supplementary file 1 [file Table1.docx]

Supplementary Table S1 The dosages of oral medication and ambulatory mean blood pressure during hospitalization.

| Hospitalization days | Nifedipine(mg) | Arotinolol(mg) | Sacubitril Valsartan(mg) | escitalopram (mg) | Tandospirone(mg) | Alprazolam(mg) | mean SBP(mmHg) | mean DBP(mmHg) |
| --- | --- | --- | --- | --- | --- | --- | --- | --- |
| 1 | 60 | 20 | 200 | 10 | 15 | 0 | 176 | 105 |
| 2 | 60 | 20 | 200 | 10 | 15 | 0 | 169 | 103 |
| 3 | 60 | 20 | 200 | 10 | 15 | 0.6 | 180 | 94.5 |
| 4 | 60 | 20 | 200 | 10 | 30 | 0.6 | 146 | 89.5 |
| 5 | 60 | 20 | 200 | 10 | 30 | 0.6 | 148 | 88 |
| 6 | 60 | 20 | 200 | 10 | 30 | 1 | 139 | 84 |
| 7 | 60 | 20 | 200 | 10 | 30 | 1 | 137.5 | 81 |
| 8 | 60 | 20 | 200 | 10 | 30 | 1 | 131.5 | 87 |
| 9 | 60 | 20 | 200 | 10 | 30 | 1 | 123 | 76.5 |
| 10 | 60 | 20 | 200 | 15 | 30 | 1 | 125.5 | 84 |
| 11 | 60 | 20 | 200 | 15 | 30 | 1 | 130 | 82 |
| 12 | 60 | 20 | 200 | 15 | 30 | 1 | 132 | 84 |
| 13 | 60 | 20 | 200 | 15 | 30 | 1 | 126 | 76 |
| 14 | 60 | 20 | 200 | 15 | 30 | 1 | 125 | 72 |

**SBP:** systolic blood pressure; **DBP:** diastolic blood pressure.

Supplementary Table S2 The dosages of oral medication during follow-up.

| follow-up (month) | Nifedipine(mg) | Arotinolol(mg) | Sacubitril Valsartan(mg) | escitalopram(mg) | Tandospirone(mg) | Alprazolam(mg) |
| --- | --- | --- | --- | --- | --- | --- |
| 1 | 60 | 20 | 200 | 15 | 30 | 0.4 |
| 3 | 30 | 10 | 200 | 15 | 30 | 0.4 |
| 6 | 30 | 10 | 100 | 10 | 20 | 0.0 |
